# Supplementary material for: Systematic Review, Meta-Analysis and Radiomics Quality Score Assessment of CT Radiomics-Based Models Predicting Tumor EGFR Mutation Status in Patients with Non-Small-Cell Lung Cancer
Source: Int J Mol Sci. 2023 Jul 14;24(14):11433. doi: 10.3390/ijms241411433 (PMC10380456; doi:10.3390/ijms241411433)
Supplement: Supplementary file 1 [file ijms-24-11433-s001.zip › ijms-2512130-supplementary.pdf]

**Supplementary Materials:**

**Table S1.** Detailed description of each article included

| Reference (APA 7th format) | First autor | Year | Number of radiomics features | Features selection method | Model | Study | Metrics/ Performance | Data base size | Ground thruth | Histology |
|---|---|---|---|---|---|---|---|---|---|---|
| Agazzi, G et al. (2021). CT texture analysis for prediction of EGFR mutational status and ALK rearrangement in patients with non-small cell lung cancer. Radiologia Medica | Agazzi, G | 2021 | Not available (only texture) | SMOTE | GBM with a nested cross-validation approach | R | Median accuracy: 81.76% [95% CI 81.45–82.06] | 84 | Segmentation: radiologists<br>EGFR: direct sequencing | Adenocarcinoma<br>Metastatisis |
| Dang, Y., et al. (2021). Clinical and radiological predictors of epidermal growth factor receptor mutation in nonsmall cell lung cancer. Journal of Applied Clinical Medical Physics | Dang, Y | 2021 | 1287 | Mann-Whitney U test<br>Spearman CC<br>RF based Boruta algorithm | Rad-Score calculated with respective weighted coefficients by using a LR | R | ROC AUC of 0.798 (95% CI: 0.664 to 0.931) | 132 | Segmentation: radiologists<br>EGFR: not mentioned (EGFR mutations identified on 4 tyrosine kinase domains (exons 18–21)) | SCC |
| Digumarthy, S. et al. (2019). Can CT radiomic analysis in NSCLC predict histology and EGFR mutation status? Medicine | Digumarthy, S | 2019 | 11 | Not mentionned | Not used (multivariable logistic regression for assessment) | R | Radiomic features (kurtosis, K2) [ROC AUC 0.656–0.713, P = .03 to .003]; radiomics, clinical & imaging features : (ROC AUC 0.890, P < .0001) | 93 | Segmentation: radiologists<br>EGFR: not mentioned (EGFR mutations identified on 4 tyrosine kinase domains (exons 18–21)) | SCC |
| Dong, Y., et al. (2022). Development and validation of novel radiomics-based nomograms for the prediction of EGFR mutations and Ki-67 proliferation index in non-small cell lung cancer. Quantitative Imaging in Medicine and Surgery | Dong, Y | 2022 | 638 | Wilkinson's ICC (intraclass correlation coefficient) | LASSO logistic regression analysis | R | ROC AUC : 0.786 (95% CI: 0.621-0.95) | 118 | Segmentation: radiologists<br>EGFR: PCR | SCC |
| He, R., et al. (2022). A Machine Learning-Based Predictive Model of Epidermal Growth Factor Mutations in Lung Adenocarcinomas. Cancers | He, R | 2022 | 622 | PCA<br>LASSO | K-nearest neighbor, RF, LGBM, SVM | R | ROC AUCs : RF 0.66±0.06; KNN 0.62±0.05; SVC = 0.67±0.04; LGBM model = 0.66±0.07 | 758 | Segmentation: radiologists<br>EGFR: direct sequencing | Adenocarcinoma |
| Hong, D., et al. (2020). Radiomics Signature as a Predictive Factor for EGFR Mutations in Advanced Lung Adenocarcinoma. Frontiers in Oncology | Hong, D. | 2020 | 396 | LASSO | LR | R | ROC AUC : 0.851 (95% CI, 0.750 to 0.951) | 201 | Segmentation: radiologists<br>EGFR: PCR | Adenocarcinoma |
| Jia, T. Y., et al. (2019). Identifying EGFR mutations in lung adenocarcinoma by noninvasive imaging using radiomics features and random forest modeling. European Radiology | Jia, T | 2019 | 440 | pvalue of each feature in the training cohort (performance driven) | EF classifiers | R | ROC AUC: 0.802. ROC AUC further improved to 0.828 by adding sex & smoking history. Se & sp: 60.6% & 85.1% at the best diagnostic decision point | 503 | Segmentation: radiologists<br>EGFR: PCR | Adenocarcinoma |

| Reference (APA 7th format) | First autor | Year | Number of radiomics features | Features selection method | Model | Study | Metrics/ Performance | Data base size | Ground thruth | Histology |
|---|---|---|---|---|---|---|---|---|---|---|
| Jiang, M., et al. (2019). Assessing EGFR gene mutation status in non-small cell lung cancer with imaging features from PET/CT. Nuclear Medicine Communications | Jiang, M | 2019 | 512 | LASSO | SVM 10-fold cross validation | R | ROC AUC of predicting results for 31 (18 for CT & 13 for PET) quantitative results 0.833 (95% CI: 0.74–0.93), counterpart to ROC AUC of outcomes for 4 qualitative features 0.731 (95% CI: 0.62–0.85). When quantitative features combining qualitative features: ROC AUC of predicting results for the whole 35 features: 0.953 (95% CI: 0.88–1) | 80 | Segmentation: radiologists<br>EGFR: PCR | SCC |
| Koyasu, S., et al. (2020). Usefulness of gradient tree boosting for predicting histological subtype and EGFR mutation status of non-small cell lung cancer on 18F FDG-PET/CT. Annals of Nuclear Medicine | Koyasu, S | 2020 | 7 type (not mentioned) | Not mentioned | RF classifiers<br>Gradient tree boosting | R | ROC AUC values: RF, single type: 0.625; XGB, single type: 0.617; RF, multiple types: 0.577; XGB, multiple types: 0.659. | 138 | Public database | SCC |
| Le, N. Q. et al. (2021). Machine learning-based radiomics signatures for egfr and kras mutations prediction in non-small-cell lung cancer. International Journal of Molecular Sciences | Le, N | 2021 | 851 | Univariate selection, RFE, Feature importance, Filter methods, F-score, GA, minimum redundancy feature selection KBest algorithm | kNN, LR, RF, XGBoos | R | ROC AUC : 0.89 | 161 | Public database | SCC |
| Li, S., et al. (2019). Radiomics for the prediction of EGFR mutation subtypes in non-small cell lung cancer. Medical Physics | Li, S | 2019 | 580 | Not mentioned | LR 5-fold cross validation | R | ROC AUC: 0.7925 | 312 | Segmentation: radiologists<br>EGFR: PCR mutations in EGFR exons 18 or 20, or simultaneous mutations in EGFR exons 19 & 21 | SCC |
| Li, X. et al. (2018). Detection of epithelial growth factor receptor (EGFR) mutations on CT images of patients with lung adenocarcinoma using radiomics and/or multi-level residual convolutionary neural networks. Journal of Thoracic Disease | Li, X | 2018 | Not mentioned | RF classifiers | Multi-level residual CNNs based modeL | R | ROC AUC : 0.74 CI 0.670 0.811 | 1010 | Segmentation: radiologists<br>EGFR: PCR | Adenocarcinoma |
| Liu, Y. et al. (2016). Radiomic Features Are Associated With EGFR Mutation Status in Lung Adenocarcinomas. Clinical Lung Cancer | Liu, Y. | 2016 | Not mentioned | Not mentioned | multiple LR | R | ROC AUC : 0.667(95% CI, 0.604-0.721) | 298 | Segmentation: radiologists<br>EGFR: PCR | Adenocarcinoma |
| Liu, Y et al. (2022). Development and Validation of Machine Learning Models to Predict Epidermal Growth Factor Receptor Mutation in Non-Small Cell Lung Cancer: A Multi-Center Retrospective Radiomics Study. Cancer Control | Liu, Y. | 2022 | 1085 | Intraclass correlation coefficient, hypothesis tests and LASSO. | LR, DT, RF, SVM | R | ROC AUC: .88, 95% CI: .75-.946 | 346 | Segmentation: cardiothoracic disease imaging physicians<br>EGFR: not mentioned | SCC; Large cell carcinoma pulmonary sarcomatoid carcinoma |

| Reference (APA 7th format) | First autor | Year | Number of radiomics features | Features selection method | Model | Study | Metrics/ Performance | Data base size | Ground thruth | Histology |
|---|---|---|---|---|---|---|---|---|---|---|
| Lu, L et al. (2020). Radiomics prediction of egfr status in lung cancer—our experience in using multiple feature extractors and the cancer imaging archive data. Tomography | Lu, L | 2020 | IBEX (1563 features) & Pyradiomics (1319 f.), & in-house software, CIFE (1160 f.) | Included in the model | KNN, SVM, LASSO, RF bagging | R | IBEX, Pyradiomics, and CIFE yielded similar areas under the ROC of 0.68, 0.67, & 0.69. However, in validation, areas under the ROC of multivariate prediction models from IBEX, Pyradiomics, & CIFE decreased to 0.54, 0.56 & 0.64 | 228 | Public database | SCC |
| Nair, J. et al. (2021). Radiogenomic Models Using Machine Learning Techniques to Predict EGFR Mutations in Non-Small Cell Lung Cancer. Canadian Association of Radiologists Journal | Nair, J | 2021 | 326 | LR | LR | R | 0.8307+/-0.07 | 50 | Segmentation: radiologists EGFR: PCR | Adenocarcinoma |
| Ninomiya, K et al. (2021). Robust radiogenomics approach to the identification of EGFR mutations among patients with NSCLC from three different countries using topologically invariant Betti numbers. PLoS ONE | Ninomiya, K | 2021 | 52 | Not mentioned | SVM 5-fold cross validation | R | 0.86(0.78–0.93 | 194 | Segmentation: radiologists EGFR: PCR | SCC |
| Rossi, G et al. (2021). Radiomic Detection of EGFR Mutations in NSCLC. Cancer Research | Rossi, G | 2021 | 104 | Variation test-restest PCA | SVM | R | ROC AUC : 0.85 | 109 | Segmentation: radiologists EGFR: PCR | SCC |
| Shiri, I., et al. (2022). Impact of feature harmonization on radiogenomics analysis: Prediction of EGFR and KRAS mutations from non-small cell lung cancer PET/CT images. Computers in Biology and Medicine | Shiri, I | 2022 | 218 | ML-based feature selection (not specified) | RF | R | ROC AUCs 0.92–0.94 for EGFR | 136 | Public database | SCC |
| Tu, W et al. (2019). Radiomics signature: A potential and incremental predictor for EGFR mutation status in NSCLC patients, comparison with CT morphology. Lung Cancer | Tu, W. | 2019 | 234 | ICCs, Mann-Whitney U, Unsupervised consensus clustering | Multivariable logistic regression | R | ROC AUC 0.775 (0.703-0.847) | 404 | Segmentation: radiologists EGFR: not mentioned | NSCLC (no precision) |
| Li, X., et al. (2019). Predictive Power of a Radiomic Signature Based on. Front Oncol | Li,X | 2019 | 38 | LASSO | LASSO | R | ROC AUC (95%CI) = 0.83 (0.68, 0.92) | 115 | Segmentation: radiologists EGFR: PCR | |
| Li, Y., et al. (2018). CT Slice Thickness and Convolution Kernel Affect Performance of a Radiomic Model for Predicting EGFR Status in Non-Small Cell Lung Cancer: A Preliminary Study. Scientific reports | LI,Y | 2018 | 1695 | SVM | CCC | R | 0.83 (0.68, 0.92) | 51 | Segmentation: radiologists EGFR: PCR | Adenocarcinoma |
| Wang, C., et al. (2022). Predicting EGFR and PD-L1 Status in NSCLC Patients Using Multitask AI System Based on CT Images. Frontiers in Immunology | Wang, C | 2022 | 1247 | LASSO | NLP | R | ROC AUCs: DL 0.906(0.885-0.930) Radiomics 0.860(0.816-0.902) Joint 0.928(0.909-0.946) | 3629 | Segmentation: Not mentioned EGFR: not mentioned | SCC |

| Reference (APA 7th format) | First autor | Year | Number of radiomics features | Features selection method | Model | Study | Metrics/ Performance | Data base size | Ground thruth | Histology |
|---|---|---|---|---|---|---|---|---|---|---|
| Yang, C et al. (2020). Application of CT radiomics features to predict the EGFR mutation status and therapeutic sensitivity to TKIs of advanced lung adenocarcinoma. Translational Cancer Research | Yang, C | 2020 | 715 | LASSO | LR 10-fold cross validation | R | ROC AUC values of the unenhanced, arterial & venous phases in the EGFR mutation status training group: 0.6713, 0.8194 & 0.8464 | 253 | Segmentation: radiologists EGFR: PCR ARMS | Adenocarcinoma |
| Yang, L., et al. (2022). PET/CT Radiomic Features: A Potential Biomarker for EGFR Mutation Status and Survival Outcome Prediction in NSCLC Patients Treated With TKIs. Frontiers in Oncology | Yang, L | 2022 | 1037 | Univariate LR analysis to select features with P< 0.05 for the subsequent analysis. Secondly, multivariate LR analysis applied to choose features closely related to different EGFR status. Informative features retained using the LASSO | SVM, DT, RF | R | ROC AUC of EGFR-WT, EGFR-19-WT, and EGFR-21-WT, with 0.881, 0.851, and 0.849, respectively, and validation ROC AUCs of 0.926, 0.805 & 0.859, respectively | 313 | Segmentation: radiologists EGFR: PCR | Adenocarcinoma |
| Yang, X., et al. (2022). Using contrast-enhanced CT and non-contrast-enhanced CT to predict EGFR mutation status in NSCLC patients—a radiomics nomogram analysis. European Radiology | Yang, X | 2022 | 1092 | Mann-Whitney U test, Spearman CC, RF based Boruta algorithm | ML methods included SVM, LR, RF, GBDT, & NBC | R | 0.850 (0.608–1.000) | 412 | Segmentation: radiologists EGFR: PCR | NSCLC (no precision) |
| Zhang, J., et al. (2020). Value of pre-therapy 18F-FDG PET/CT radiomics in predicting EGFR mutation status in patients with non-small cell lung cancer. European Journal of Nuclear Medicine and Molecular Imaging | Zhang, J | 2020 | 45 | LASSO | RF | R | 0.87 (95%CI [0.79–0.95]) | 248 | Segmentation: radiologists EGFR: PCR | Adenocarcinoma |
| Zhang, L., et al. (2018). Quantitative Biomarkers for Prediction of Epidermal Growth Factor Receptor Mutation in Non-Small Cell Lung Cancer. Translational Oncology | Zhang, L. | 2028 | 485 | LASSO | A multivariable logistic regression | R | AUXC wkith and without clinical feartures0.62 to 0.85 | 180 | Segmentation: radiologists EGFR: PCR | Adenocarcinoma: 48.6% (68/140) of cases; SCC: 38.6% (54/140); others: 12.8% (18/140) |

*CCC=Concordance correlation coefficient; CIFE=Columbia Image Feature Extractor; DT=decision tree; Feature Extractor; GBDT=gradient boosting decision tree; GBM=Generalized boosted regression model; ML=Machine learning; NBC=naive Bayesian classification; LASSO=Least absolute shrinkage and selection operator; LR= logistic regression; R=retrospective; RF=Random forest; SCC=Squamous cell carcinoma; Se=Sensitivity; SMOTE=Synthetic minority oversampling technique; Sp=specificity; ; SVM=support vector machine; RFE=Recursive feature elimination; ROC=receiver operating characteristic curve; PCA=Principal component analysis*
